# Practical Hygiene, as It Relates to a Proper Water Supply — the Defective Sewerage of Cities and Towns, Hospitals and Asylums*This paper was read before the Wisconsin State Medical Society, at the annual meeting in Milwaukee, June 5, 1879.

**Published:** 1879-08

**Authors:** J. N. DeHart


### Article II.

### Practical Hygiene, As it Relates to a Proper Water Supply — The Defective Sewerage of Cities and Towns, Hospitals and Asylums.* By J. N. DeHart, m.d.

The importance of the observance of the laws of hygiene, as they relate to a proper supply of pure water, and the imperfect sewerage of our large cities and towns, hospitals and asylums, is only receiving, at the present day, but a small share of the public attention which they so urgently demand.

This paper will allude to some of the impurities that are found in water, as revealed by the microscope, and the diseases produced by them, together with the bad effects resulting from defective sewerage.

The want of a good supply of pure water for domestic purposes leads to many bad results, not only in a private residence, where the wants of a family are not very great, but also in large communities where many persons reside, and in hospitals and asylums where many hundred patients are collected together for the treatment of diseases. It not only gives rise to impurities of all kinds, but the person and clothing are not properly cleansed, or are washed repeatedly in the same water; water for cooking purposes is used scantily; houses are not cleansed, streets become dirty, and sewers are clogged; and in these various ways a lack of a proper supply of pure water gives rise to impurities in the air itself. As a result of the facts just stated, we have an im-

---

*This paper was read before the Wisconsin State Medical Society, at the annual meeting in Milwaukee, June 5, 1879.

pairment of the health of the population. The deficiency of water in the cleansing of sewers causes the development of typhoid fever and choleraic diarrhœa, and our records show that epidemics of the latter have been checked by a heavy rainfall. The supply of water is a matter of the most urgent necessity, where persons are undergoing great muscular efforts, and it is very important and proper that it should be furnished in small quantities and quite frequently given.

It may be true that water containing a small quantity of organic matter may be used for a short time with but slight ill effects, but its continued use has caused the development and spread of diseases in many communities.

In some of these cases, very little attention has been paid, or inquiry made into the state of health of those using the water, and that most fallacious of all evidence, *a general impression*, without a careful collection of facts, has often been the only ground on which the opinion has been based.

This much is certain, that as correct investigations are carried on, and careful microscopic examinations are made, a continually increasing class of cases is found to be connected with the use of impure water, and it seems only reasonable to infer that a more rigid inquiry will further prove the frequency and importance of this mode of origin of some diseases; animal organic matter, especially when of fæcal origan, vegetable organic matter, when derived from marshes, and some salts are the principal noxious ingredients of the dangerous substances; the suspended animal matter and especially fæcal matters, are probably the worst.

This is shown in outbreaks of diarrhœa and typhoid fever. This fact makes the chemical examination of the color and turbidity so important. The thoroughly dissolved organic matter appears less hurtful.

Probably, also, the more recent the fæcal contaminations, the more injurious, since it has been observed, that the most poisonous attacks occur near wells into which, after slow percolation for some time, a sudden gush of sewage has taken place.

Animal matters forming fatty acids produce salts which though not oxidizing into nitrous and nitric acid, are as hurtful as the more oxidizable substances.

There have been a variety of opinions expressed in regard to the action of minerals in water, which is used for drinking and domestic purposes. While there are some mineral substances, such as sodium chloride or carbonate, or calcium carbonate, which within certain limits appear to do no harm, yet there are other mineral substances, such as calcium, and magnesian sulphates and chlorides, and calcium nitrate, which are undoubtedly injurious to many persons; a combination of impurities, and especially the co-existence of organic matter and calcium sulphate is hurtful; this has been proven by the analysis of water which contains them and which has produced disease. As far as at present known, the existence of infusoria of various kinds is not injurious, though their presence may indicate the existence of an abundance of organic impurities.

That impurities of water do cause diseases by their action upon the membranes, with which they come in contact, may be proven by an enumeration of some affections of the alimentary mucous membranes, viz.: dyspepsia, diarrhœa, dysentery, etc.

In the first mentioned disease, the use of large and frequent doses of magnesian salts, calcium sulphate and chloride in water may produce it.

The latter diseases may be caused by suspended mineral substances of clay, mud, etc., as the waters of the Mississippi and Missouri rivers,* also the waters of the Ganges and other rivers, at certain periods of the year, especially to persons unaccustomed to their use. The scales of mica are said to have caused diarrhœa among the inhabitants of Dhumsala, India; also suspended animal and fæcal matters and vegetable substances have been the cause of sudden attacks of diarrhœa.

The former and latter when washed off the ground by heavy rains into streams and rivers which supply cities and towns, have caused an epidemic of catarrh of the alimentary canal, as is frequently observed in this country.

In the summer of 1876, owing to the very slight rain fall, the Croton river which supplies the city of New York with water, became so nearly dry that there was great fear that the city would

* Hammond's Hygiene.

lack a sufficient supply of water for ordinary use. During the following September there was a very heavy rain fall, and the vast water sheds which supply this river, were completely drenched ; the decaying animal and vegetable matters which had been exposed to a long drought were carried down to the receiving reservoirs, and immediately following this heavy rain-fall, there was an outbreak of typhoid and malarial fevers within the hospitals of the city, as well as an endemic of diarrhœa and dysentery.

It is a well established fact that dissolved and putrescent animal organic matter, to the amount of 30 to 50 Ctm. per liter, may produce diarrhœa. Fæcal gases will cause catarrh of the alimentary canal, especially if organic matter be also present.

This was observed during the invasion of Mexico by the French, in 1861 and '62. This army suffered from a peculiar dyspepsia and diarrhœa at Orijaba, which was attended with the disengagement of gas and eructation of food, after meals. This gas was sulphureted hydrogen, and was traced to the use of water from sulphurous and alkaline springs. The symptoms of poisoning by water contaminated by sewage, are frequently similar to those already mentioned, viz., diarrhœa and dysentery, and sometimes irritations of the kidneys, liver, spleen and lungs. Pinel observed this fact 20 years since in the wards of Salpétrière, and Duchaulet noticed that the water furnished the patients at St. Lazare contained a large proportion of sulphate of lime, and other purgatives, which caused frequent attacks of diarrhœa. Nitrate of lime has a similar effect. Brackish water will also affect the mucous membrane lining the alimentary tract.

Occasionally animal organic matter acts in an indirect way by producing nitrates which act on metals. In illustration of this fact, a German physician, who was called to see persons who were supposed to have been drinking impure water, and became sick, found on examining the water, that it contained a considerable amount of copper. The cylinder of the pump was made of copper, and this had combined with some organic matter in the water and caused them to be poisoned.

The effect of drinking impure water is also frequently shown in the dysentery which develops among the soldiers who encamp in the vicinity of swamps and marshes. In the late civil war, the Union army, while passing through the Chickahominy swamps of Virginia, suffered very much from dysentery. The water which they drank was mostly surface water. When a well was sunk, it seldom exceeded ten or twelve feet in depth, and the water always had a brackish taste, and frequently emitted a foul odor, especially if it had been allowed to stand in a vessel for a few hours.

The same impurities which produce dysentery are closely allied to those which develop diarrhœa. The fact that dysenteric stools can propagate disease, render it quite probable that, as in the case of typhoid fever and cholera, the accidental passage of dysenteric evacuation into wells may have some share in spreading the disease.

To the late Dr. Snow, of England, are we indebted for valuable researches relating to the dissemination of some diseases by the use of water for domestic purposes.

Malarious fevers are very prevalent in the vicinity of marshes.

It was Hippocrates who first asserted that those persons who drink the water of marshes suffer from an enlargement of the spleen. Other writers have not only confirmed this fact, but claimed that it generated fevers.

One very important circumstance is the rapidity of the development of malarious disease, and its fatality when introduced by water. We have the same result in the case of diarrhœa and dysentery. Either the fever-producing cause must be in larger quantity in the water, or what is equally probable, must be more readily taken up into the circulation and carried to the spleen, than when the same cause enters by way of the lungs. That typhoid fever may be developed by the use of impure water, as well as impure air, is a theory of quite recent origin; although as far back as the middle of the last century, one German physician advanced a similar opinion.

Dr. Flint in his clinical reports on continued fevers in 1852, alludes to an outbreak of typhoid fever, which occurred in North Boston, Erie county, N. Y., in 1843, and this was attributed to the contamination of the water by the contents of a sewer passing into it. That water may be the medium of propagating typhoid fever, has been proven by the evidence of many English physicians and German authorities, such as Profs. Budd, Jenner, Wilkenson, Barclay, Seaton, Latham, Muller, Richter, Burkhardt and Wohlial.

Profs. Clark, Metcalf, Loomis and others of this country, have fully corroborated the views of these distinguished English and German physicians.

One English authority states that in cases where the poison is conveyed by water, the infection seems to be much more certain; and he has reason to think that the period of incubation is materially shortened.

The question may sometimes be asked, what is the proportion of cases of typhoid fever that are disseminated by the use of impure water, as compared with those that are caused by the inhalation of impure air? While no positive answer has yet been given to this question, yet it has been observed that when the dates of attacks are given, the incubation period in those who have drunk impure water appears to be very short; while it is probable that it takes from eight to ten days after the typhoid poison has entered with the air before the early malaise comes on; yet in some cases of typhoid fever produced by water, only two or three days were observed to elapse before the symptoms are marked. Will decomposing sewage in water produce typhoid fever, or must the evacuations of a typhoid patient pass into it?

This is part of the larger and important question relating to the origin and propagation of specific poisons. How often, everything is attributed to fæcal matters merely, without searching for other causes, which may be as potent as this, in the development of typhoid.

A recent number of the Berlin *Klinische Wochenschrift* contains an account of five cases in which infected water was the undoubted medium of contamination. A miller living on the bank of a small stream, was attacked with typhoid fever. His excreta were thrown daily into a small pond which was connected with the stream by a ditch. This man recovered, but seven weeks afterward, his brother, who alone nursed him, became ill with symptoms of typhoid. The discharge from the bowels were very profuse, and were, as in the other case, thrown also into the pond.

A few days afterwards four laborers, who were employed in a forge about one mile distant, were attacked with typhoid, and the medical attendant, anxious to discover the source of the mischief, found that all of these men obtained their drinking water from the stream in question, there being no well accessible. Another case also occurred, the patient being a farm laborer, who worked in a meadow near the forge, and also drank water from the same rivulet. No case of typhoid had occurred in this vicinity for a long time previously, and all the circumstances were in favor of the supposition that the disease had been transmitted through the medium of the water. There was no evidence as to the source of infection of the first patient, and the period of incubation was extremely long, particularly in the case of the brother. The history of this small epidemic tends to support Dr. W. Budd's view, that a specific agent must be present, for the production of typhoid fever. No doubt the water in the rivulet alluded to, often contained more or less ordinary fæcal matter. May we not infer from this, that the excreta of typhoid patients should be thoroughly disinfected, and when possible, as in country districts, should be deeply buried in places far removed from any source of water supply? The London *Lancet* has quite a full account of an outbreak of typhoid fever, in Glasgow, Scotland, and the source of infection was traced to the supply of milk from certain dairies; after the death of two prominent citizens, the sanitary officers of that city began an investigation which traced the contaminated milk supply to two large dairies. Though these circumstances were known, it was reserved for another medical officer conclusively to show that the two dairies in question were fed by a constant supply of water from an adjoining farm, where typhoid patients had lived for several weeks previous; and that the sanitary arrangements of this farm were very defective; that the excreta of the patients were thrown into a channel into which the water ran that supplied the two dairies.

The water with which the dairy vessels were washed was obobtained from the same supply. One hundred and sixty-three cases of typhoid were developed among the customers of these two dairies, and the death rate was about 10 per cent. Of seven students at the university who had the fever, three died. Should not these melancholy facts lead to the proper and periodic inspection of town dairies and their country feeders? It is not a very pleasant thought to realize that disease may unwittingly be brought to our doorsteps every morning through the single medium of the most essential of all nourishing fluids for family use!

In one of our Western asylums, which had been supplied with water from an artesian well and several cisterns, the rapid increase of the population required a more abundant supply, and an iron pipe was laid to connect with an inland lake. This lake was fed by a river which received its supply from an extensive marsh. The sewage of the asylum, State university and many private residences was discharged into this lake.

The use of the artesian well and cistern waters were discontinued in the wards of the asylum about May, 1, 1877, and a very abundant supply of water was obtained from the lake.

No malarial nor typhoid fevers had developed in the wards of the asylum, while the artesian and cistern waters were in use.

About one month after the lake water was introduced, a case of well marked typhoid was developed in one of the male wards. The patient died on the morning of the 21st day from the attack, having had a hæmorrhage from the bowels in the evening of the 19th day.

Other cases of intermittent, remittent, typho-malarial and typhoid fever continued to develop during the summer and fall, and also during the winter months. The fever appeared both in male and female wards, and patients and employes were alike attacked by them.

Some cases of typhoid were accompanied with severe gastric symptoms, which seemed to baffle all remedies. In one case, that of an employé, the gastric symptoms were very severe, and for about ten days both medicine and nourishment were rejected. There was also a persistent diarrhœa, with marked tympanitis. Subsultus was present, and during the third week there was a wild delirium. A hæmorrhagic discharge from the bowels occurred in the evening of the 19th day, which had been preceded by a severe chill. He died on the morning of the 21st day.

In many cases of intermittent and remittent, as well as typhomalarial fevers, the patients had epistaxis and pharyngitis. All the cases of typhoid had a diarrhœa, and those of the male patients who had hæmorrhagic discharges from the bowels, died. Petechia was present in all cases of typhoid. From June, 1877, till June, 1878, there were in the male wards 30 cases of intermittent, 11 cases of remittent; 4 cases of typho-malarial; 12 cases of typhoid. Total, 57. Population of male wards about 188.

Two patients died of typhoid, and all the others recovered.

In the female wards, there were about the same number of cases, but a slight increase of deaths. From June, 1878, until August, 1878, there were fifty cases. Six of these were well-marked cases of typhoid, and two of them died. At first it was thought that this endemic of malarial fevers was due to the marsh, which approached to within one mile of the asylum, at the point where the river emptied into the lake, but the continuance of the fever during the winter months, and its rapid development in the spring, naturally led to a microscopic and chemical examination of the water of the lake. From the large quantity of sewage matter that was being daily discharged into the lake from the asylum (and this was impregnated with the fever poison from the excreta of the patients), and the several sewers of the city of Madison, it was not surprising to find organic matter in the water. The sewer from the asylum discharged its contents into the lake about 900 feet from the end of the water pipe.

The water examined with the microscope was drawn from the tanks at 6½ a. m., and after the water cocks had been open half an hour. It was pumped into the tanks at 5 a. m. The following, infusoria, animalcules, protozoa, rhizopods, rotifers, vibriones, and many microscopic forms of vegetable life, together with organic matter, were found in great abundance.

Animalcules. — Tricoda Lynceus, 4; Vorticella Nebulefera, 5; Zoospores, 2. Protozoa.—Actinophrys Sal, 8; Amœba, 15; Rhizopoda.—Cucella Acuminata, 10. Entomostraca.—Daphnia Pulex, 13. Rotifera.—Rotifera Vulgaris, 16. Vibriones.—Vibriones, 14; Oscillaria Lawes, 7. Tubular prolongation of pigment cells, 21.

Microscopic Forms of Vegetable Life.

Desmidiacea.—Pediastrum Granulatum, 1; Fungi, 12; Puccianii Grammi, 18. Diatomacea.—Surrilla Constricta, 6; Surrilla Striatula, 11;

Mastoglia Smithii, 9; Cocconema Lanceolatum, 3. Organic matter, 20. Decaying vegetable matter, 17. Volvex Globiator, 19.

Infusoria or infusory animalcules are found almost universally in infusions of organic matter.

Vegetable matter, portions of woody fiber, small bits of roots and parenchyma were found. Some of the vegetable tissue was more or less decomposed, and dark, opaque, structureless masses were seen. Sewage matters, having a darkish brown or reddish color, and very frequently seen in globular masses, and thus distinguishable from the flattened vegetable matter were found in considerable quantity. Nitric acid applied to the sediment ncreased the redness of the deposit of organic matter. This is Hassel's test for organic matter, who first recognized these little ocherous masses, and thought them to be muscular fiber tinged with bile.

Vibriones or microzyomes are the terms applied to small points or jointed rods, which frequently move with great rapidity, and then become quiet or motionless. Some make a distinction between them, while others consider the terms as synonymous.

Their presence indicates the existence of an organic, carbonaceous substance, a nitrogenous substance and a phosphate. Bacteria is a term also applied to the above. This latter may originally exist in the water or be introduced. Burden-Sanderson claims that they cannot be introduced from the air, and that they are sometimes so small as not to be detected by the highest microscopic powers, or by the test of Tyndall with the electric beam. While it has not been conclusively shown that bacteria or vibriones are in themselves hurtful (although some observers think they are), yet their presence indicate the co-existence of certain organic substances and putrefaction, and the latter is always dangerous.

Rhizopoda and amœba may indicate, like bacteria, the existence of putrefying substances.

The mere presence of diatoms should not condemn water as being impure on account of their presence. Entomostraca (water flea) are quite abundant in the spring, and being found in so many waters, are not regarded as dangerous.

The rotifera (wheel animalcules) are frequently found, and although not considered as dangerous, yet their existence indicates an impurity of the water. The great number of microscopic objects found in water have led observers to look first for mineral substances, and ascertain by chemical tests their origin and quantity; then to examine the water, by aid of the microscope, for bacteria, fungi, amœba and ova, and small worms and leeches. These latter indicate an impurity of the water, which makes it dangerous for drinking or culinary purposes.

The various forms or kinds of infusoria, diatoms and algæ are important in connection with microscopic evidence of decaying vegetable matters, and with chemical tests showing much dissolved organic impurity in water.

